# Epigenetic, environmental, and sex-specific programming of long-term health in Assisted Reproductive Technologies–conceived offspring^†^

**DOI:** 10.1093/biolre/ioag137

**Published:** 2026-07-01

**Authors:** Eric A Rhon-Calderon, Cassidy N Hemphill, Marisa S Bartolomei

**Affiliations:** Department of Cell and Developmental Biology, Perelman School of Medicine, Epigenetics Institute, University of Pennsylvania, Philadelphia, PA, USA; Department of Cell and Developmental Biology, Perelman School of Medicine, Epigenetics Institute, University of Pennsylvania, Philadelphia, PA, USA; Department of Cell and Developmental Biology, Perelman School of Medicine, Epigenetics Institute, University of Pennsylvania, Philadelphia, PA, USA; Center for Women's Health and Reproductive Medicine, University of Pennsylvania Perelman School of Medicine, Philadelphia, PA, USA

**Keywords:** ART, development, epigenetics, environment, sex

## Abstract

Assisted Reproductive Technologies (ART) are non-coital methods of conception widely used to address infertility. These procedures include conventional in vitro fertilization (IVF), intracytoplasmic sperm injection, gamete and embryo cryopreservation, trophectoderm biopsy for pre-implantation genetic testing, and newer mitochondrial replacement techniques. Since the first IVF birth in 1978, the global use of ART has increased rapidly, contributing to more than 13 million births worldwide. Although ongoing technological advances have improved safety and increased pregnancy and live-birth rates, ART has also been associated with adverse placental and fetal outcomes, some of which persist into later life and, in certain cases, across generations. The mechanisms underlying these outcomes are not fully understood, but studies suggest that altered epigenetic reprogramming, environmental influences, and sex-specific factors are major contributors. In this review, we examine how long-term health outcomes in ART-conceived offspring are shaped by the interplay of epigenetic mechanisms, environmental influences, and sex-specific responses in both humans and mice.

## Teaser

In vitro fertilization–conceived offspring have exhibited epigenetic and metabolic adverse outcomes compared to naturally conceived offspring.

## Introduction

Assisted Reproductive Technologies (ART) are medical treatments used to facilitate the fertilization of eggs in individuals or couples experiencing infertility, delayed childbearing, or requiring donor gametes, including same-sex couples [1]. ART includes any external manipulation of gametes or embryos to achieve a successful pregnancy, with in vitro fertilization (IVF) and intracytoplasmic sperm injection (ICSI) currently being the most widely used techniques [2]. Since the first IVF birth in 1978, which was achieved using eggs collected from a natural cycle through a more invasive retrieval procedure, ART has advanced substantially, improving pregnancy and live-birth rates while also becoming more accessible worldwide [1].

IVF currently accounts for approximately one-third of all ART cycles globally, while ICSI has become the predominant method regardless of a male infertility diagnosis [2]. Both techniques share several procedural steps but differ in how fertilization is achieved. In IVF, fertilization occurs ex vivo when oocytes collected from hormonally stimulated females are co-incubated with donor sperm and cultured under optimized conditions until the blastocyst stage [3]. In contrast, although ICSI also uses superovulated oocytes, fertilization is accomplished by directly injecting a single sperm head into the oocyte, bypassing several natural barriers involved in gamete interaction [4]. Currently, both IVF and ICSI also include cryopreservation of gametes or embryos, either before use or after fertilization, to allow delayed embryo transfer [3, 4]. Additionally, newer technologies, such as trophectoderm biopsy for pre-implantation genetic testing of aneuploidies or specific genetic disorders, have been added to traditional ART cycles with the goal of further improving pregnancy success and overall offspring health [5, 6].

**Figure 1:**
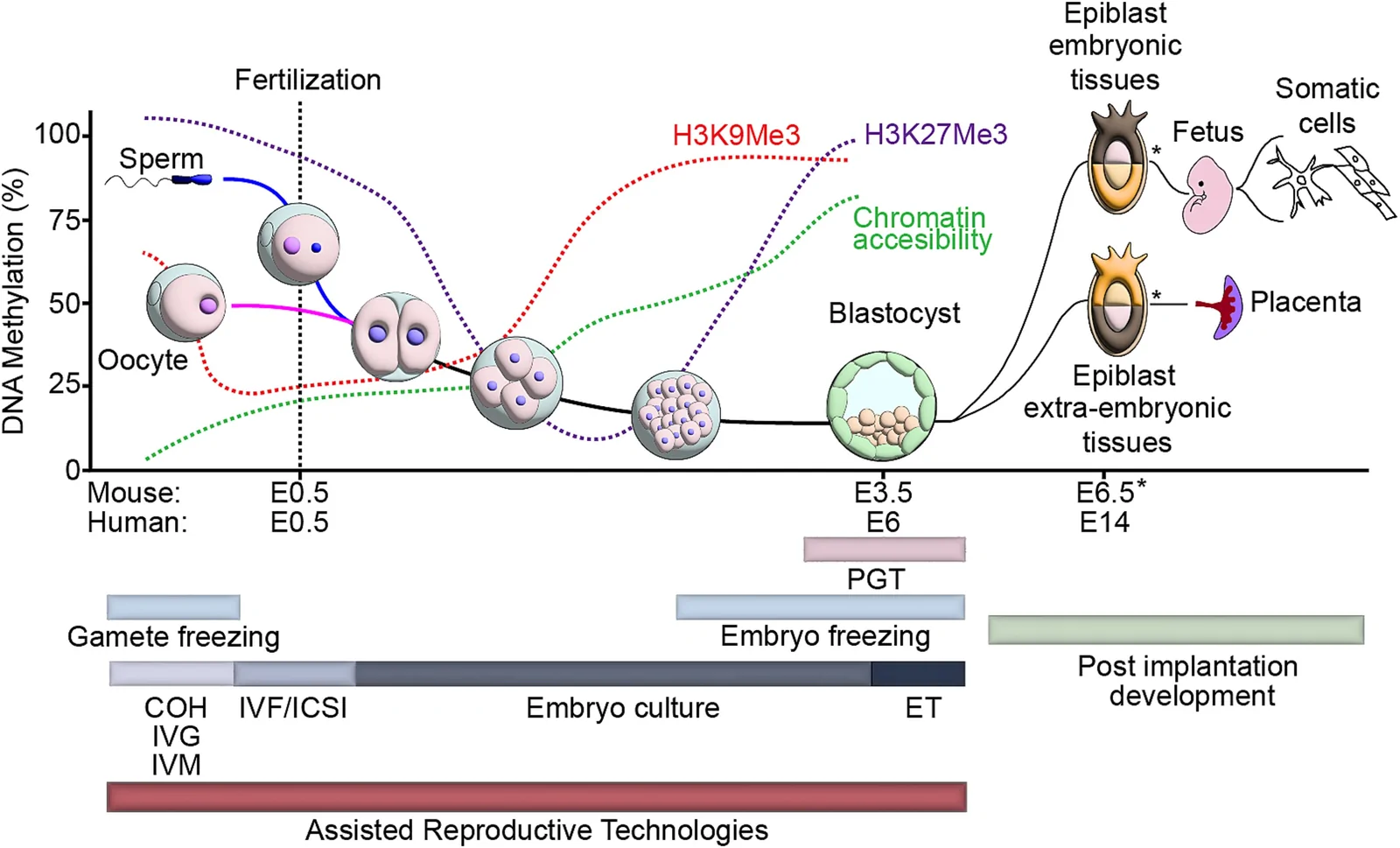
Epigenetic reprogramming in humans and mouse. The schematic shows changes in DNA methylation in oocytes, sperm, and the developing embryo, together with the dynamics of H3K9me3, H3K27me3, and chromatin accessibility. ART procedures occur during this sensitive window of epigenetic reprogramming. The post-implantation egg cylinder shown at E6.5 is mouse-specific and is marked with an asterisk; in mice, the extraembryonic ectoderm derives from polar trophectoderm, whereas human embryos do not form an egg cylinder or extraembryonic ectoderm and instead develop a bilaminar embryonic disc. E, embryonic day; COH, controlled ovarian hyperstimulation; IVG, in vitro gametogenesis; IVM, in vitro maturation; IVF, in vitro fertilization; ICSI, intracytoplasmic sperm injection; PGT, pre-implantation genetic testing; ET, embryo transfer. Adapted from [25, 26].

Although ART procedures are generally considered safe, they have been associated with a range of adverse outcomes affecting the gestational mother, as well as the fetus and placenta [7–9]. Studies from our laboratory and others have shown that these outcomes are largely driven by epigenetic dysregulation during the pre-implantation period, a developmental window characterized by extensive genome-wide DNA methylation erasure and re-establishment, as well as dynamic reprogramming of histone post-translational modifications. During this period, gametes and embryos are directly manipulated and maintained in artificial culture environments, rendering them particularly vulnerable to perturbations in chromatin organization, genomic imprinting maintenance (i.e. monoallelic, parental-specific origin expression [10, 11]) and transcriptional regulation. These effects are further shaped by the composition of the culture environment and by sex-specific epigenetic responses, collectively contributing to persistent molecular and physiological alterations in ART-conceived offspring [12–20] (Fig. 1). Importantly, mechanistic insights have also emerged from other mammalian systems [21, 22]. Studies using other animal models, such as cattle, sheep, and pigs, have revealed ART-associated developmental abnormalities, including large offspring syndrome, placental dysregulation, and imprinting disturbances, providing additional evidence that early embryonic manipulation can disrupt conserved epigenetic and developmental pathways across species [22–24].

In this review, we summarize and discuss the long-term health outcomes identified in ART-conceived populations, in both mice and humans, and highlight the major drivers underlying these changes, including disrupted epigenetic mechanisms, environmental factors such as culture conditions and maternal influences, and the impact of sex on developmental and postnatal phenotypes. Finally, we outline the current state of the field, identify key gaps that require attention, and discuss future directions for improving ART safety and understanding its biological consequences.

## ART and pre-implantation development

**Figure 2:**
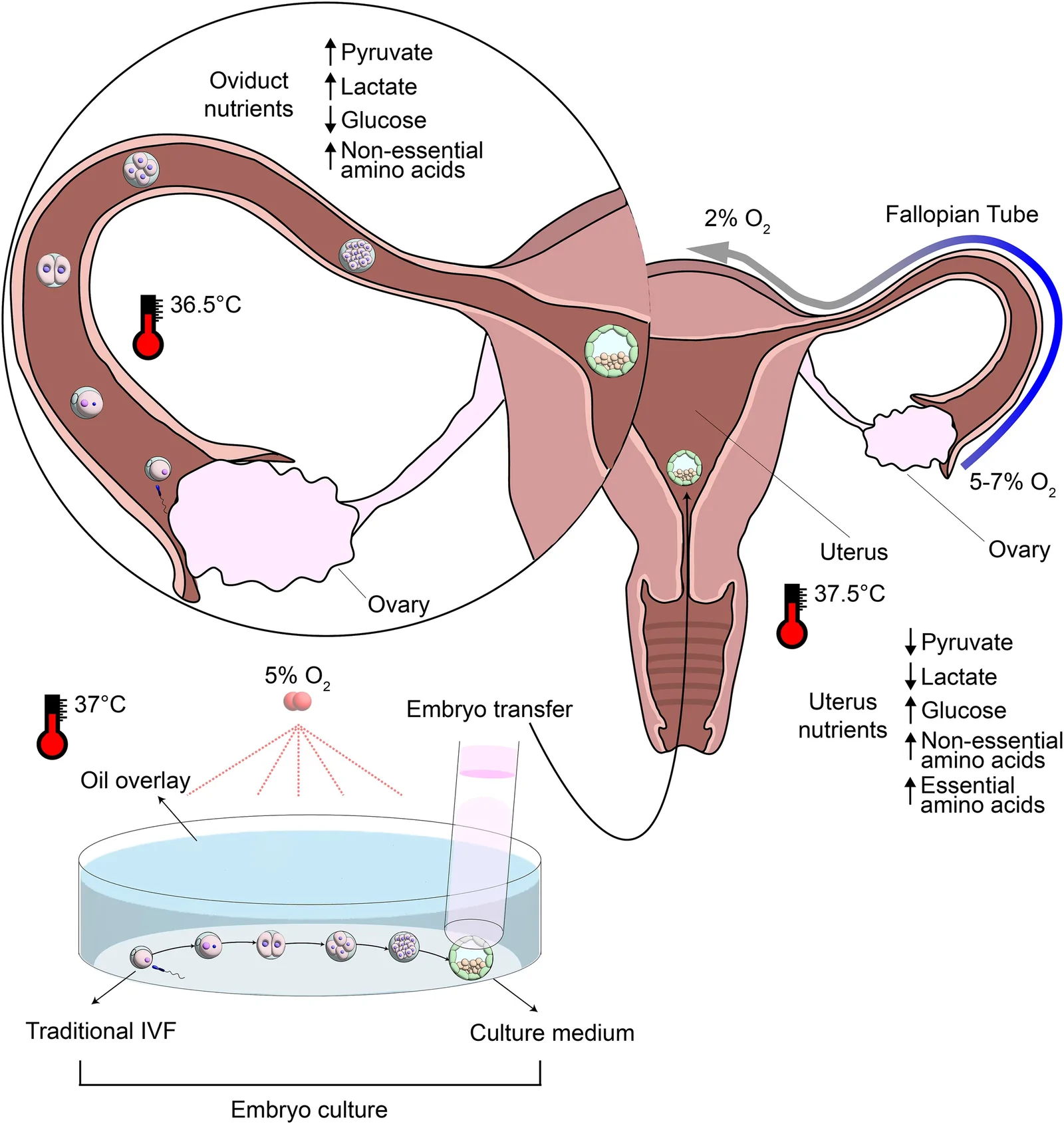
In vivo versus in vitro pre-implantation embryo environments. Diagram comparing the physiologic conditions experienced by embryos during natural conception and traditional IVF. In vivo (left), embryos move from the ovary through the fallopian tube to the uterus, encountering dynamic gradients of oxygen (∼5–7% O₂ in the oviduct to ~2% O₂ in the uterus), temperature, and nutrient availability, with higher pyruvate and lactate early and increased glucose and amino acids later. In contrast, IVF embryos are cultured ex vivo (bottom left to middle) under static conditions (∼37°C, typically ~5% O₂) in defined media before embryo transfer, bypassing the native oviductal–uterine transitions that shape early metabolic and developmental programming. Adapted from [28].

Since the first successful IVF pregnancy, a landmark achievement in reproductive medicine, ART have undergone continuous refinement to optimize gamete manipulation and embryo culture conditions to approximate the physiological environment of the female reproductive tract [27] (Fig. 2). Foundational studies in animal models, particularly in mice, were instrumental in defining the culture parameters, metabolic requirements, and laboratory practices that now underpin modern ART protocols. These advances have substantially improved embryo viability and pregnancy success rates [26]; however, they have also increased the duration and complexity of embryo exposure to artificial conditions during pre-implantation development. In this section, we examine key physical, chemical, and metabolic factors that must be precisely regulated to support normal embryonic development and implantation, while highlighting how perturbations in these factors may contribute to adverse outcomes in ART-conceived pregnancies.

Preimplantation development is characterized by extensive molecular and epigenetic reprogramming that establishes the foundation for subsequent embryonic and extraembryonic development [29]. Following fertilization, the embryonic genome undergoes global DNA demethylation accompanied by dynamic remodeling of histone post-translational modifications and large-scale chromatin reorganization, processes that are tightly coordinated with early cleavage divisions and lineage specification [30] (Fig. 1). Although most of the genome is reset during this period, a small but functionally critical subset of genes, known as imprinted genes, escapes global demethylation and maintains parent-of-origin–specific DNA methylation patterns throughout development [31]. These imprints are established in the germline and preserved after fertilization by imprinting control regions (ICRs), which function as cis-regulatory elements that coordinate allele-specific gene expression across imprinted domains [32]. This reprogramming phase is highly sensitive to environmental cues, including metabolic substrates, oxygen tension, and extracellular signals, rendering the early embryo particularly vulnerable to perturbations introduced by ART [9].

Disruption of DNA methylation or chromatin regulation at ICRs can lead to aberrant monoallelic expression, resulting in congenital imprinting disorders as well as long-term developmental and metabolic abnormalities, with downstream consequences for placental formation, fetal growth, and health across the lifespan [33]. Here we analyzed two major contributors to the ART adverse associated outcomes: the embryo culture and the oxygen tension.

### Embryo culture

Embryo culture is one of the most extensively studied components of ART, with the primary goal of optimizing embryonic development to increase successful pregnancy and live birth rates [34–38]. However, recent evidence has also identified embryo culture conditions as a major contributor to the adverse outcomes observed in ART-conceived offspring [13, 36, 38–43]. Here, we review how the composition of culture media and the duration of in vitro culture influence embryonic development and may contribute to downstream health outcomes.

**Figure 3:**
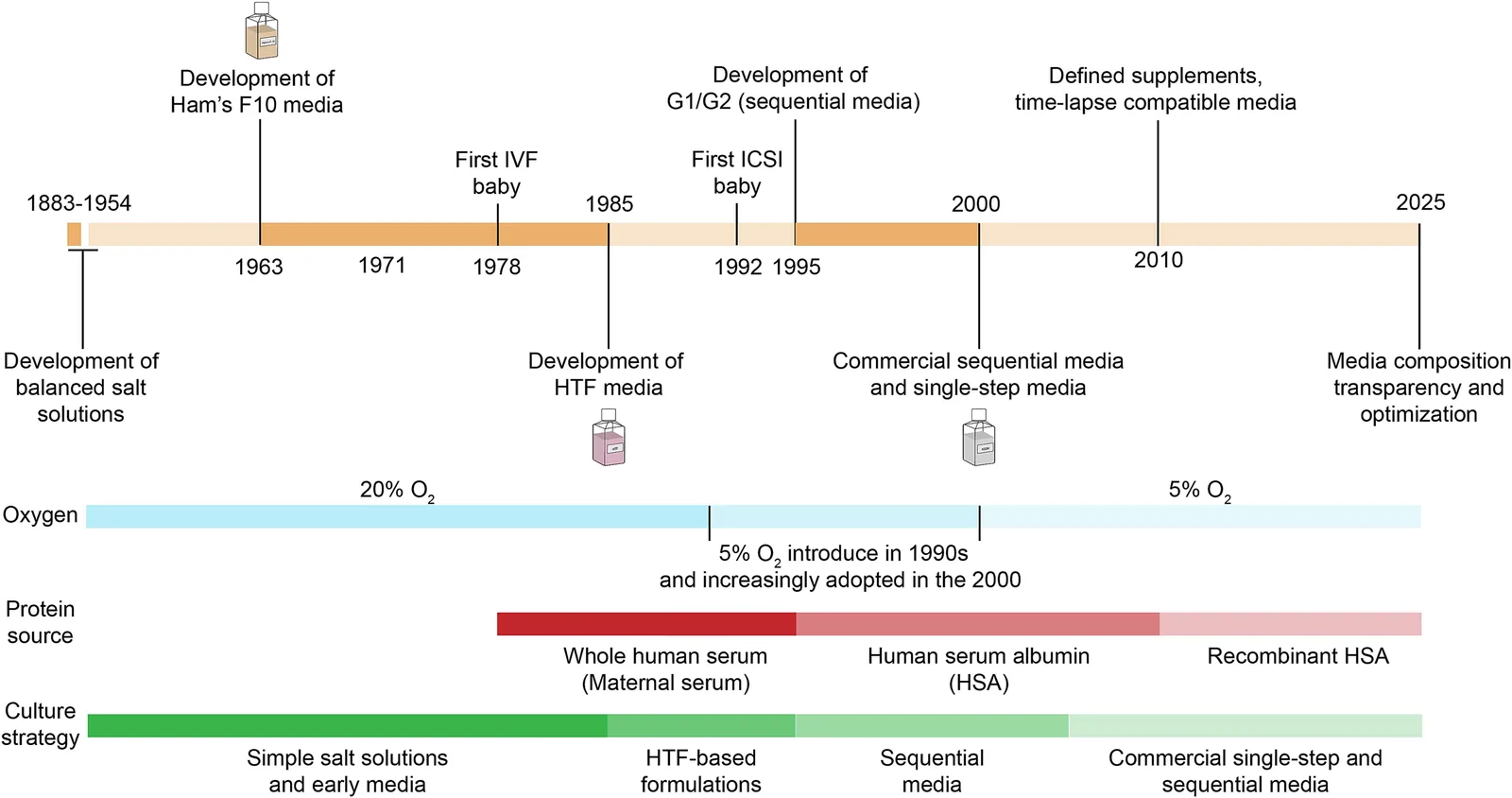
Evolution of human embryo culture systems in IVF. Schematic overview of major historical changes in human embryo culture, including the transition from balanced salt solutions and early media to HTF-based formulations, G1/G2 sequential media, and later commercial sequential and single-step media. The figure also summarizes changes in protein supplementation, from whole human or maternal serum to human serum albumin and recombinant HSA, and the gradual shift from atmospheric oxygen to low-oxygen culture. Low oxygen was introduced by pioneering clinics in the 1990s and became increasingly adopted during the 2000s; therefore, the timeline represents broad trends rather than universal implementation dates. Mouse-specific media, such as KSOM, are not included. Current concerns include variability in commercial media composition and the need for greater transparency and evidence-based optimization of culture conditions.

The culture medium serves as the microenvironment for in vitro embryo development [41]; therefore, the composition and concentration of each component must be carefully selected and rigorously tested to minimize cellular and metabolic stress during early development. Although advancements in culture media formulations have improved embryo viability and developmental outcomes, current systems still fall short of fully recapitulating the dynamic and finely regulated environment of the female reproductive tract (Fig. 3). As a result, even optimized media cannot completely prevent the physiological and epigenetic perturbations associated with in vitro development. As such, both experimental and clinical studies demonstrate that variations in nutrient composition, amino acid turnover, growth factors, buffering systems, and metabolic substrates can alter developmental trajectories, affect blastocyst quality, and modify the epigenetic landscape during key windows of reprogramming [34, 39, 41, 44, 45].

The culture medium shapes developmental outcomes by altering the metabolic and signaling milieu that governs early cell fate decisions and epigenetic programming [41, 46–50]; unlike the dynamic nutrient and oxygen gradients experienced in vivo, in vitro culture exposes embryos to static metabolite concentrations that can influence mitochondrial function, redox balance, one-carbon metabolism, and chromatin-modifying enzyme activity [10, 24, 25, 47, 51–54]. Even subtle perturbations in these processes can disrupt imprinting maintenance and lineage specification, with lasting consequences for placental development, fetal growth, and pregnancy outcomes.

The duration of embryo culture has also changed over time, largely driven by evolving clinical priorities and technological capabilities. Early IVF protocols favored relatively short culture periods, with embryos typically transferred at the cleavage stage due to limited in vitro developmental competence and suboptimal culture conditions [38, 55]. As culture media, incubator systems, and laboratory practices improved, extended culture to the blastocyst stage became increasingly feasible, allowing for improved embryo self-selection, better synchronization with endometrial receptivity, and higher implantation rates [38, 56, 57]. Nevertheless, extended culture duration, currently to the blastocyst stage in vitro, has been associated in some reports with altered imprinting, modified placental development, and differences in birthweight [35]. Concerns regarding prolonged in vitro exposure during critical windows of epigenetic reprogramming and lineage specification prompted reevaluation of extended culture in some contexts, particularly as evidence emerged linking blastocyst culture with altered placental development and perinatal outcomes [57]. For example, Vrooman et al. demonstrated in a mouse IVF model that extending embryo culture to the blastocyst stage has a pronounced impact on both fetal and placental health and, importantly, induces widespread alterations in DNA methylation patterns [13].

In humans, embryo culture strategies have evolved with increasing understanding of pre-implantation metabolic requirements, shifting from sequential culture systems to single-step media [58]. Sequential media were originally designed to mimic the changing in vivo environments of the oviduct and uterus, with early cleavage-stage embryos relying primarily on pyruvate and lactate and limited glucose utilization, followed by increased glucose uptake, amino acid turnover, and oxidative metabolism at the blastocyst stage [58]. Accordingly, sequential formulations provided stage-specific nutrient compositions, whereas modern single-step media contain a balanced mixture of energy substrates, amino acids, vitamins, lipids, and antioxidants sufficient to support development from fertilization through blastocyst formation without media exchange. The transition to single step medium reflects efforts to reduce embryo handling and environmental fluctuations. Thus, embryo culture medium does not simply support pre-implantation development; it actively shapes the biochemical and epigenetic landscape that programs fetal and placental physiology. More recently, the widespread adoption of trophectoderm biopsy for pre-implantation genetic testing has again required extended culture to the blastocyst stage [59, 60], reinforcing a clinical tradeoff between enhanced embryo selection and increased duration of in vitro manipulation.

Together, these shifts underscore how changes in culture duration have been shaped by competing goals of maximizing implantation success while minimizing potential developmental perturbations [56, 61]. In summary, continued research aimed at refining embryo culture conditions is essential for reducing ART-associated adverse outcomes and improving the long-term health of ART-conceived offspring.

### Oxygen tension

In ART, embryos develop in a controlled in vitro environment for several days, during which oxygen tension is a critical parameter influencing cellular metabolism, developmental competence, and epigenetic integrity. To more closely approximate in vivo conditions, clinics aim to culture embryos at oxygen concentrations like those found in the female reproductive tract, where physiological oxygen levels typically range between 2–8% [62, 63]. Historically, most clinics cultured embryos at atmospheric oxygen levels (~20%), which are substantially higher than those experienced in the oviduct and uterus [64] (Fig. 2 and 3). Numerous studies in mice, cattle, and other mammalian species have demonstrated that atmospheric oxygen concentrations are detrimental to embryo quality, fetal development, and placental formation [39].

**Figure 4:**
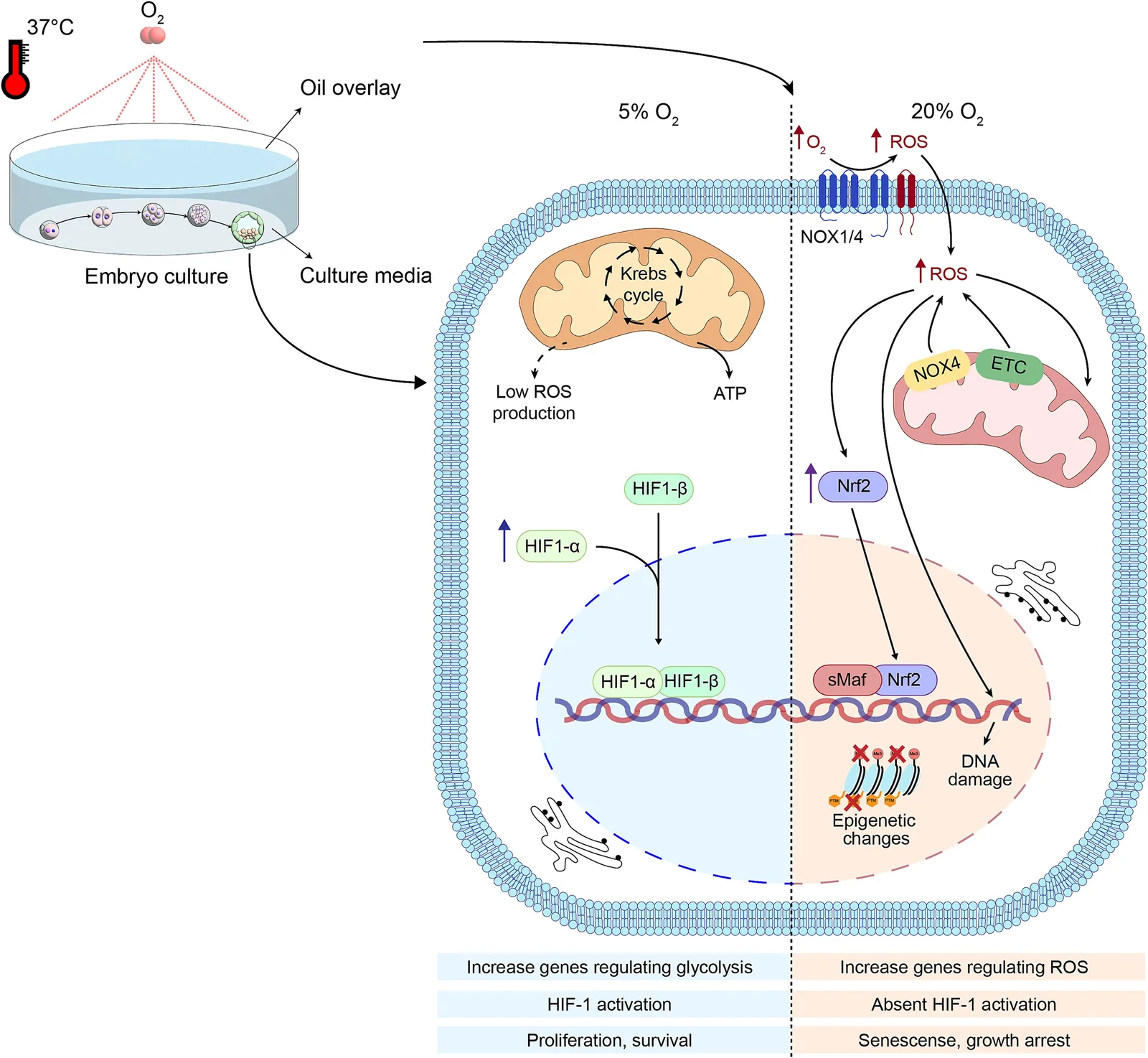
Oxygen tension–dependent metabolic and redox signaling during pre-implantation embryo culture. Schematic illustrating how oxygen concentration during in vitro embryo culture influences mitochondrial metabolism, ROS production, and downstream transcriptional and epigenetic responses. Left: low oxygen conditions (5% O₂) traditionally used in IVF, right: atmospheric oxygen (20% O₂) [65, 66].

Elevated oxygen tension during in vitro embryo culture shifts the embryonic redox and metabolic state toward oxidative stress by increasing mitochondrial and NADPH oxidase–derived reactive oxygen species (ROS). Exposure to atmospheric oxygen (20% O₂) enhances ROS production through the mitochondrial electron transport chain and NOX family enzymes, disrupting mitochondrial homeostasis and suppressing hypoxia-inducible factor-1 (HIF-1) activation (Fig. 4). Excess ROS under high-oxygen conditions activates oxidative stress–responsive pathways, including NRF2 signaling, and alters redox-sensitive gene expression programs. In contrast, physiologic low-oxygen conditions (5% O₂), now commonly used for IVF, limit ROS generation, preserve mitochondrial function, and promote HIF-1–dependent metabolic programming.

Importantly, oxidative stress during the pre-implantation period coincides with a critical window of epigenetic reprogramming, characterized by global DNA demethylation followed by de novo methylation, resetting of histone modifications, and large-scale chromatin remodeling. Increased ROS can directly and indirectly perturb these processes by altering the activity of oxygen- and metabolite-dependent chromatin-modifying enzymes, including TET DNA demethylases and Jumonji C domain–containing histone demethylases [66–69], as well as DNA methyltransferases. In parallel, oxidative stress can shift key metabolic cofactors, such as the SAM/SAH ratio and α-ketoglutarate availability, further compromising proper establishment and maintenance of imprinting marks and lineage-specific epigenetic states [29, 70–74]. Collectively, these oxygen-driven disruptions are associated with DNA damage, aberrant epigenetic patterning, altered trophoblast differentiation, and impaired placental development, with lasting consequences for offspring growth and long-term health [75, 76].

Together, these concerns prompted a shift toward culture under reduced oxygen tensions, typically around 5%, which more closely resemble oviductal and uterine physiology (Fig. 2). Subsequent clinical and experimental studies consistently reported improved blastocyst formation, enhanced embryo viability, and higher pregnancy rates under low-oxygen conditions. For example, Whitten et al. were among the first to describe the benefits of culturing mouse embryos at 5% oxygen, reporting increased blastocyst formation and higher cell counts per blastocyst [45]. Nevertheless, measurements of intrauterine oxygen tension across multiple species, including rats, humans, rabbits, and non-human primates, indicate that physiological oxygen concentrations are often closer to 2% [62, 63, 77–79], suggesting that even current “low-oxygen” in vitro systems remain too elevated compared to the female reproductive tract (Fig. 2). More recently, Nguyen et al. demonstrated that a sequential oxygen regimen, 7% oxygen during the first three days of culture followed by 2% oxygen until the blastocyst stage, increased blastocyst formation rates and accelerated cell divisions [80]. However, these studies did not include embryo transfer or implantation analyses, leaving unresolved whether reduced or sequential oxygen tensions improve not only pre-implantation development but also post-implantation outcomes, placental function, and long-term offspring health.

In summary, oxygen tension is a critical factor during embryo development, 20% oxygen induces oxidative stress, disrupts mitochondrial function, and interferes with epigenetic reprogramming during the highly sensitive pre-implantation period. In contrast, lowering oxygen concentrations to 5% improves blastocyst development, cell allocation, and overall embryo viability. Although current low-oxygen systems typically use 5% oxygen, emerging data suggest that physiological uterine oxygen levels are closer to 2%, indicating that further optimization may be necessary.

## ART adverse health outcomes

**Figure 5:**
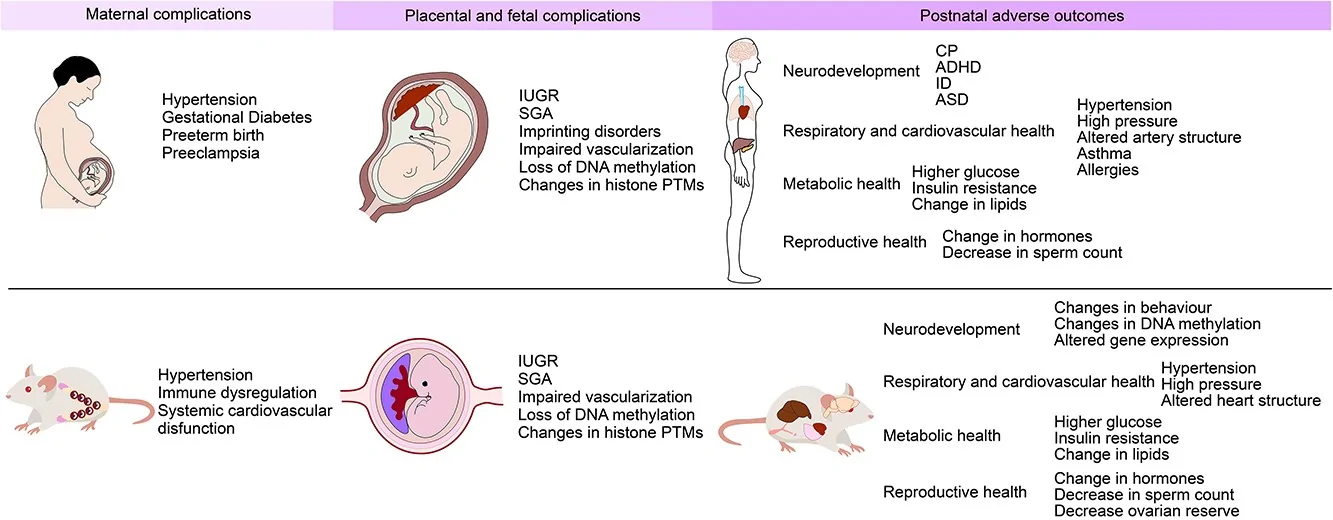
Adverse outcomes associated with ART in humans and mice. Overview of maternal, placental or fetal, and postnatal complications linked to ART, shown for humans (upper panel) and mouse models (lower panel). The mouse model may not fully translate in magnitude or clinical impact to human risk. Nevertheless, these experimental models provide important mechanistic insight into how ART related manipulations during early development may influence biological pathways that are relevant to human ART populations. Maternal effects include hypertensive and metabolic disorders, immune dysregulation, and cardiovascular dysfunction. Placental and fetal outcomes include intrauterine growth restriction (IUGR) and small for gestational age (SGA), impaired vascularization, and epigenetic alterations. Postnatally, ART offspring exhibit adverse neurodevelopmental outcomes, including CP, ADHD, ID, and ASD, as well as metabolic, cardiovascular, respiratory, and reproductive dysfunctions, highlighting conserved biological pathways that may be affected across species.

ART has been associated with a range of adverse outcomes affecting both the fetus and the placenta, with long-term health implications [12, 14, 17, 18, 40, 81–86]. Numerous studies in both humans and mice have shown that ART can disrupt normal development as early as the pre-implantation period. Furthermore, alterations in placental function, including impaired vascularization and nutrient transport, have been reported, along with fetal growth abnormalities and metabolic perturbations in ART-conceived offspring compared with naturally conceived controls [12, 14–16, 19, 87–94]. These early developmental deviations have been associated with postnatal phenotypes such as cardiac and metabolic dysfunction, reproductive impairments, and accelerated aging [81–83, 95–98]. Importantly, several studies in both humans and animal models demonstrate that some ART-associated changes are persistent, irreversible, and capable of propagating across generations, highlighting the sensitivity of the early embryo to procedural and environmental influences (Fig. 5). Such findings underscore the need to better understand the mechanisms through which ART perturbs developmental programming, particularly the roles of epigenetic reprogramming, culture environment, and sex-specific responses, therefore safer and more physiologically aligned reproductive technologies can be developed.

### ART and adverse placenta outcomes

The placenta is a transient but vital organ that supplies nutrients and oxygen to the fetus, removes metabolic waste, and regulates maternal immune tolerance [99, 100]. Proper placental development is essential for fetal growth, and its dysfunction, placental insufficiency, can lead to adverse developmental outcomes such as fetal growth restriction (FGR), which is associated with increased perinatal mortality and long-term cardiovascular, metabolic, and neurodevelopmental impairments [99, 100].

Although placental structure varies among species, its core functions are conserved across mammals [101]. Limited access to early human placentas makes animal models, particularly mice, essential for studying extraembryonic development [102]. Human and mouse placentas share a broadly comparable organization consisting of decidual, junctional, and exchange zones, and both depend on hemochorial placentation, which allows direct contact between trophoblasts and maternal blood. Important differences remain, for example, deeper trophoblast invasion and early dominance of the placenta over the yolk sac in humans [103], endocrine functions such as hCG and placental estrogen production, and differing numbers of syncytial layers, but conserved cellular and molecular pathways still make the mouse a strong model for investigating placental biology and disease.

Normal placental development requires precise gene regulation, including epigenetic mechanisms that direct lineage specification, trophoblast differentiation, and expression of key growth-related genes such as imprinted genes [104]. Studies in both humans and mice have demonstrated the major contribution of epigenetics to placental formation and function, as well as the pathological consequences of epigenetic dysregulation at any developmental stage [89, 105, 106]. For example, deletion of placental-specific *Igf2* in mice leads to reduced placental size and fetal growth restriction [107]. In human placentas, reduced *DNMT1* expression and global DNA hypomethylation have been observed in early pregnancy losses compared to term placentas [108, 109]. Also, in intrauterine growth restriction (IUGR), imprinted genes such as *PHLDA2* and *CDKN1C* are upregulated, whereas *MEG3*, *GATM*, *ZAC1*, *GNAS*, *MEST*, *IGF2*, and *Syncytin-1* are downregulated, with corresponding effects on fetal birthweight [110–112]. Many of these expression changes correlate with altered DNA methylation at ICRs as well as at non-imprinted regulatory elements [112].

Other genomic features also undergo epigenetic disruption during placental pathologies, including repetitive elements such as LINE-1, which show decreased methylation in placentas from low-birthweight infants [113]. Although mice do not develop true preeclampsia, experimental models demonstrate that altering DNA methylation at imprinted, non-imprinted, or repetitive loci can induce preeclampsia-like phenotypes, including hypertension and impaired spiral artery remodeling [15, 114, 115]. These data highlight the sensitivity of the placenta to disruptions in the epigenome.

ART has been associated, in both humans and mice, with multiple placental dysfunctions, including placenta accreta, placenta previa, aberrant trophoblast invasion, altered vascular remodeling, and an increased risk of preeclampsia [114]. These abnormalities reduce placental efficiency, compromise nutrient and oxygen exchange, and ultimately impair fetal growth, predisposing offspring to long-term metabolic and cardiovascular disease. As suggested above (Fig. 1), ART-induced placental defects likely originate from disruptions in epigenetic reprogramming during the pre-implantation period [12, 13, 87] in both humans and mice.

In ART mouse pregnancies, placental weight is frequently increased at term, while fetal weight is reduced, a pattern consistent with placental inefficiency [88]. In mouse models, IVF and related procedures similarly produce enlarged placentas with smaller fetuses, reflecting altered placental development, impaired vascularization, and dysregulated trophoblast differentiation [12, 13, 15, 16, 87]. These findings underscore that ART-related perturbations during early embryogenesis can lead to lasting structural and functional defects in the placenta. This phenotype parallels observations in human pregnancy complications such as preeclampsia and in conditions associated with poor fetal growth, including stillbirth and advanced maternal age.

In addition to DNA methylation defects, emerging evidence shows that ART also alters post-translational histone modifications (PTMs) in the placenta, further contributing to abnormal placental development and function [89]. Early embryogenesis is a critical window during which histone marks such as H3K4me3, H3K27me3, H3K9me3, and H3K36me3 are dynamically reprogrammed to regulate trophoblast lineage commitment, enhancer accessibility, and placental gene expression [116]. ART procedures, including superovulation, in vitro fertilization, embryo culture, and embryo transfer, can disrupt the establishment or maintenance of these histone landscapes. Studies in mice have shown reduced H3K27me3 imprinting domains in extraembryonic tissues following IVF, leading to dysregulation of noncanonical imprinted genes that control placental growth [117]. Similarly, alterations in H3K4me3 and H3K9me3 have been observed at promoters and repetitive elements, affecting trophoblast proliferation, invasion, and labyrinth formation [118]. Additionally, changes in H3K4me3 in extraembryonic tissue is a major cause of implantation failure and abnormal placental development of IVF embryos [119]. These histone abnormalities interact with DNA methylation defects to amplify transcriptional instability, ultimately impairing placental efficiency and fetal development.

### ART and Fetal adverse outcomes

One of the first indications that IVF and ART may disrupt epigenetic regulation came from observations of a higher-than-expected incidence of imprinting disorders in ART conceived children, many of which originate from loss of DNA methylation at ICRs of imprinted gene clusters [120–122]. For example, early studies reported that IVF conceived children had up to a tenfold increased risk of Beckwith–Wiedemann syndrome (BWS) compared with naturally conceived children [123]. Other imprinting disorders, including Silver–Russell syndrome (SRS), Angelman syndrome, and Prader–Willi syndrome (PWS), have also been reported at elevated frequencies in ART pregnancies. More recently, population-based analyses in Sweden suggest that certain ART procedures may contribute to a small but measurable increase in the risk of specific imprinting disorders. Children conceived using ICSI combined with cryopreserved embryos have been reported to have a modestly elevated risk of BWS, PWS, and SRS, independent of parental background factors including infertility [124]. Although these syndromes remain rare and the absolute risk is low, their association with ART highlights the sensitivity of epigenetic reprogramming during early embryonic development and underscores the importance of continued investigation into how specific ART procedures may influence imprint maintenance and epigenetic stability [123, 124].

In addition to an increased rate of imprinting disorders, in humans ART has been associated with higher rates of adverse perinatal outcomes compared to naturally conceived offspring. For example, ART singletons have increased rates of preterm birth, low birth weight, and small for gestational age (SGA), as well as a higher risk of stillbirth, particularly before 28 weeks of gestation [125]. Moreover, in humans, a large cohort study comparing ART-conceived singletons with those conceived spontaneously reported an elevated risk of major birth defects across multiple organ systems, including the central nervous system; eye; ear, face, and neck; cardiovascular system; gastrointestinal tract; urinary system; and musculoskeletal system, with congenital heart defects being the most frequently observed anomalies [126, 127].

Importantly, the risk of birth defects appears to vary by ART procedure. ICSI has been associated with a higher incidence of specific anomalies compared to conventional IVF. For instance, cohort data from Australia demonstrated increased rates of certain congenital malformations in ICSI-conceived children [128], and another study found a higher frequency of urogenital defects in infants conceived using ICSI compared to IVF [129].

In summary, the use of ART has been associated with an increased risk of congenital anomalies compared with natural conception, with the magnitude of risk varying by procedure type and appearing strongest for ICSI. These defects are thought to arise from multiple, non–mutually exclusive mechanisms, including epigenetic dysregulation during early embryonic cell-fate specification, disruption of normal genome and imprinting reprogramming, and aberrant placental development, all of which can influence organogenesis and fetal growth. However, substantial controversy remains regarding the causal contribution of ART itself, as most individuals undergoing ART do so because of underlying infertility. It has therefore been proposed that parental subfertility, genetic factors, or associated comorbidities may independently predispose offspring to congenital anomalies, complicating the attribution of risk solely to ART procedures [130–132]. In addition, long-term follow-up studies of ART-conceived individuals have examined broader health outcomes beyond congenital anomalies, including cancer risk in childhood and young adulthood, although the absolute risk appears low and findings remain under active investigation [133]. This interpretation contrasts with findings from mouse models, in which ART procedures can be experimentally isolated from infertility and reproducibly induce congenital, placental, and metabolic abnormalities. Interestingly, in fertile mouse strains, ART has been shown to cause loss of DNA methylation at ICRs, accompanied by consistent epigenetic and phenotypic alterations across studies, including the altered placental phenotype, supporting a direct role for ART-related manipulations in driving adverse developmental outcomes.

### ART and maternal adverse outcomes

ART pregnancies have increased gradually and steadily since the first successful IVF procedure; however, this rise has been accompanied by an increased rate of adverse pregnancy outcomes [134]. Previous studies have shown that ART-conceived pregnancies are associated with a higher risk of gestational diabetes mellitus (GDM), gestational hypertension and its progression to preeclampsia, placental abnormalities, and preterm birth [135–138]. It remains unclear whether these increased risks arise primarily from ART procedures themselves or from the underlying infertility of the parents. Some studies, such as Stern et al., suggest that ART procedures may contribute more significantly to obstetric and neonatal risks than infertility alone [139], whereas others, emphasize that maternal and paternal factors, such as infertility diagnosis, age, hormonal disbalances, or pre-existing comorbidities, also play major roles [140]. Thus, the relative contributions of ART-specific interventions versus underlying parental characteristics remain an area of active investigation. A large Chinese cohort comparing ART pregnancies with naturally conceived pregnancies demonstrated significantly higher risks of GDM, preeclampsia, moderate or severe anemia, hepatic and thyroid disorders, preterm birth, placenta previa, postpartum hemorrhage, cesarean delivery, stillbirth, and abnormal fetal development [141].

Multiple studies have concluded that the increased rate of adverse pregnancy outcomes observed in ART cycles is influenced, at least in part, by procedural components inherent to ART. Superovulation protocols expose the endometrium to supraphysiologic hormone levels that may impair decidualization. Embryo culture conditions, including oxygen tension, temperature stability, pH, and media composition, can influence embryo quality and subsequent placental development. Additionally, the fertilization method (e.g. ICSI vs. conventional IVF) may differentially affect epigenetic programming at fertilization. Despite these associations, current evidence remains insufficient to fully delineate the mechanistic pathways through which ART increases the risk of obstetric complications, and further research is needed to separate procedural effects from underlying infertility and maternal health factors [141–144].

One approach to elucidate the relative contributions of underlying infertility versus ART-related procedures to maternal outcomes is the use of mouse models. As noted above, ART paradigms in mice recapitulate many key human outcomes with remarkable fidelity, including alterations in placental development, fetal growth trajectories, epigenetic regulation, and other long-term health phenotypes in offspring. Importantly, mouse models allow ART procedures to be performed in fertile females, thereby isolating the effects of gamete and embryo manipulation from confounding factors associated with subfertility [145]. Although maternal outcomes associated with ART have been far less extensively studied than offspring phenotypes, there is a need of animal models to evaluate the impact of ART procedures on maternal physiology during pregnancy. In mice, given the well-documented effects of ART on placental development, fetal growth, and offspring metabolic health, it is plausible that maternal physiological adaptations to pregnancy are also altered. Collectively, this gap in knowledge highlights a critical opportunity to leverage mouse ART models to mechanistically dissect how ART-related manipulations influence not only offspring development but also maternal health during and after pregnancy, independent of underlying infertility.

### ART long-term health outcomes in the offspring

ART have been associated with adverse long term health outcomes in both male and female offspring. Some outcomes have been attributed to various procedures used in ART, including ovarian hyperstimulation [146], method of fertilization [147], in vitro culture of gametes and embryos, genetic testing [148], vitrification and warming of gametes and embryos [149], and even embryo transfer. Consequently, there is an increased interest in identifying adverse outcomes, with the intention to improve these technologies and make it safer for both fetus and mom. Nevertheless, the accelerated adoption of these technologies has not allowed the scientific community to conduct robust studies to address the risks. Here we will discuss the impact of ART on different systems and how they vary depending on offspring sex.

#### ART and Neurodevelopment

Neurodevelopmental outcomes and potential manifestations later in life are increasingly the focus of attention. The developing nervous system, especially during the earliest stages of embryogenesis, is highly vulnerable to a range of endogenous and environmental influences. Disruptions during this period can contribute to neurodevelopmental conditions such as attention-deficit/hyperactivity disorder (ADHD), intellectual disability (ID), autism spectrum disorder (ASD), and cerebral palsy (CP) [150–155]. These disorders may arise from perturbations in normal neural development that may result from genetic predisposition, environmental exposures, immune-mediated mechanisms, or interactions among these factors. Increasing evidence now highlights epigenetic dysregulation, like aberrant DNA methylation, altered histone modifications, and changes in chromatin organization, a critical mechanistic pathway linking early developmental exposures to altered neurodevelopmental risk [156].

Because of the early exposures involved in ART, the procedures may modify neurodevelopmental trajectories. The artificial in vitro environment can influence metabolic pathways required to produce essential epigenetic cofactors, including S-adenosylmethionine, acetyl-CoA, and α-ketoglutarate, that govern the activity of DNA and histone-modifying enzymes [157]. Perturbations in these metabolic and epigenetic processes during the cleavage and blastocyst stages may alter neuronal lineage commitment, disrupt synaptic maturation, and interfere with the formation of brain-specific chromatin architecture [158–160]. Furthermore, ART-associated alterations in placental development, may secondarily affect fetal brain development by compromising nutrient and oxygen transport, modifying hormonal signaling, and increasing vulnerability to inflammatory cues.

Emerging molecular evidence demonstrates that ART conception is associated with distinct DNA methylation signatures and altered expression of genes involved in neurodevelopment when compared to naturally conceived newborns, suggesting that ART procedures can influence epigenetic pathways integral to brain maturation [161]. Complementary findings in mouse models further support this idea: ART-derived mice show persistent alterations in DNA methylation and histone modifications in neural tissues, as well as changes in the expression of genes involved in synaptic development, neuronal differentiation, and stress responsivity, providing mechanistic insight into how early embryonic perturbations may shape brain function later in life [162, 163]. In contrast, findings from large epidemiological studies in humans offer a more nuanced perspective. Data from the Nordic registry, one of the most comprehensive cohorts of IVF-conceived individuals, indicate that overall neurodevelopmental outcomes are largely comparable between ART and non-ART singletons [164]. ART-conceived children displayed slightly increased adjusted risks of learning impairments, motor coordination difficulties, and a modest elevation in the likelihood of ASD and ADHD diagnoses.

Sex could be another possible contributor to the observed changes. The contribution of sex in neurodevelopmental outcomes following ART is an emerging but largely poorly understood area. As stated before, large registries and cohort studies generally report minimal differences in neurodevelopmental disorders between ART and naturally conceived offspring and consequently do not identify strong ART-by-sex interactions. The modest increases in ASD or ADHD observed in some studies appear to mirror the baseline male predominance of these conditions rather than reflect ART-specific sex effects [164].

In conclusion, mouse and other animal studies provide mechanistic evidence that ART can influence neurodevelopmental programming through altered metabolic signaling, epigenetic regulation, placental function, and neural gene expression. However, human data remain more nuanced, with large registry studies generally showing comparable neurodevelopmental outcomes among ART and non-ART singletons, despite modest increases in some diagnoses such as ASD, ADHD, learning impairments, or motor difficulties. These associations should be interpreted cautiously because absolute risks are generally low, effect sizes are modest, and it remains unclear whether mechanisms identified in animal models translate into clinically significant neurodevelopmental disease in humans. Longitudinal, sex-stratified studies integrating placental, epigenomic, neurodevelopmental, and clinical endpoints are needed to define the real-world impact of ART on brain health across the lifespan.

#### ART and cardiovascular health

The effects of ART on cardiovascular development in offspring remain uncertain, particularly in humans. Some experimental studies in mice provide important mechanistic insights about the impact of ART in cardiovascular health of offspring. For example, ART has been shown to alter the expression of genes involved in RNA synthesis, RNA processing, and cardiovascular development [165]. Another study demonstrated the dysregulation of 42 epigenetic modifiers in ART offspring heart, with several imprinted genes critical for cardiac growth and metabolism downregulated, suggesting disruption of imprint maintenance during early development. Beyond transcriptional and epigenetic changes, a few functional abnormalities have also been observed. Watkins et al. reported that embryo culture increases blood pressure, a marker of higher risk of hypertension, in mouse ART-offspring with higher impact on females [166]. A study in mice showed that ART-conceived offspring exhibited altered cardiac morphology, including ventricular wall thinning and reduced cardiomyocyte proliferation, along with impaired cardiac relaxation and early signs of diastolic dysfunction [95]. Mitochondrial defects, such as diminished respiratory capacity, increased reactive oxygen species production, and altered mitochondrial DNA copy number, have also been reported in the hearts of ART offspring, suggesting disrupted metabolic programming. Collectively, these studies demonstrate that ART can perturb cardiac development at the molecular, structural, and functional levels in mice, providing a mechanistic framework for understanding potential cardiovascular vulnerabilities observed in ART-conceived humans.

In humans, several epidemiological investigations have reported an elevated risk of cardiovascular defects, like congenital heart defects (CHDs) among ART-conceived children. A meta-analysis found that ART offspring are approximately 50% more likely to present CHDs than naturally conceived infants, while another cohort study identified a 1.38-fold increased risk among IVF-conceived children [98]. Cardiac remodeling and functional alterations have also been documented in ART-conceived fetuses compared with spontaneously conceived counterparts, and some of these differences appear to persist into infancy, childhood, and adolescence. Another study in humans reported reduced flow-mediated dilation of the brachial artery and increased carotid intima–media thickness, which are markers consistent with early endothelial dysfunction [167]. Similarly, a US pilot study observed greater arterial stiffness and impaired endothelial responses in ART-conceived children [130, 168]. Although these studies involve relatively small cohorts, their findings indicate that ART may influence vascular physiology and early cardiovascular development.

Other studies in humans have raised concerns regarding blood pressure regulation, with a meta-analysis reporting higher rates of elevated blood pressure in ART-conceived children [165]. The etiology of these cardiovascular alterations remains unclear, but one proposed mechanism is that ART disrupts cardiovascular epigenetic programming during critical windows in which key regulators of cardiac and vascular development are established. Nevertheless, parental characteristics such as advanced maternal age, higher BMI, metabolic disorders, and the underlying causes of infertility may also contribute significantly to these observations, making it challenging to distinguish ART-specific effects from those associated with parental or perinatal factors.

ART has also been associated with sex-specific developmental vulnerabilities. In mouse models, an ART-related skewing of the sex ratio has been attributed to peri-implantation developmental defects that disproportionately affect female embryos [169]. These defects have been linked to impaired imprinted X-chromosome inactivation, suggesting that early epigenetic disturbances may differentially impact embryo viability by sex. Given that females typically exhibit a more favorable cardiovascular risk profile at younger ages, such sex-specific developmental perturbations could influence ART-associated cardiovascular trajectories.

In conclusion, mouse studies provide mechanistic evidence that ART can alter cardiovascular programming, including cardiac gene expression, imprinting, mitochondrial function, vascular physiology, and blood pressure regulation. Human studies suggest possible associations with congenital heart defects, endothelial dysfunction, arterial stiffness, and elevated blood pressure, but these findings should be interpreted cautiously because absolute risks are low and effect sizes are generally modest. It remains unclear whether animal mechanisms translate into clinically significant cardiovascular disease in ART-conceived individuals. Longitudinal studies with careful control for parental and infertility-related factors are needed to define the real-world cardiovascular impact of ART.

#### ART and immune health

Recent studies have suggested that ART is associated with an increased incidence of immune-related alterations in offspring, in both humans and animal models. In mice, ART has been linked to impaired immune function, including reduced vaccine responsiveness, diminished activation of T helper type 2 (Th2) pathways, and significantly decreased IFN-γ/IL-4 and IgG2a/IgG1 ratios compared to naturally conceived controls [170–172]. Additional mouse studies have reported reduced thymus size, altered thymocyte maturation, impaired regulatory T-cell (Treg) development, and changes in natural killer (NK) cell activity, indicating that ART may disrupt both innate and adaptive immunity. ART-conceived mice also show altered macrophage polarization and cytokine profiles, including reduced IL-10 and increased pro-inflammatory cytokines such as TNF-α, suggestive of chronic low-grade inflammation. These alterations extend to the placenta, where ART has been associated with dysregulated expression of immune-related genes, abnormal leukocyte recruitment, and disrupted cytokine signaling networks [14, 173].

Human studies similarly report broad immune perturbations in ART-conceived offspring. Epidemiological analyses have found that ART-conceived children have a higher risk of immune-related disorders, including allergies, asthma, pneumonia, recurrent respiratory infections, and other pulmonary conditions [174, 175]. ART offspring also exhibit altered cord blood immune cell profiles, including differences in monocyte, dendritic cell, and T-cell subsets, as well as changes in cytokine responses at birth. Some studies have reported an increased risk of autoimmune diseases, including type 1 diabetes, juvenile idiopathic arthritis, and autoimmune thyroiditis, though findings vary across populations. Moreover, ART-conceived newborns show altered immunoglobulin production, disrupted NK cell function, and increased susceptibility to infectious disease–related hospitalizations in early childhood [170]. Together, these findings suggest that ART may influence multiple dimensions of immune system maturation, from innate immune readiness to acquired immune regulation.

Despite these observations, the precise mechanisms underlying immune alterations in ART offspring remain incompletely understood. The developing immune system is highly sensitive to its intrauterine and early postnatal environment, and several factors may contribute, including parental health status, infertility-related conditions, and the supraphysiological hormonal environment associated with ART cycles. Nonetheless, a growing body of evidence points to epigenetic perturbations during pre-implantation development as a major contributor to immune dysfunction [176]. These disruptions are particularly relevant because immune lineage specification, cytokine programming, and long-term immune regulatory networks are established during windows of extensive epigenetic reprogramming. While these findings highlight the immune system as a sensitive target of ART-related perturbations, their overall magnitude, mechanisms, and long-term clinical relevance remain incompletely defined. Larger mechanistic and longitudinal studies are needed to determine how ART shapes immune health across childhood and into adulthood.

In conclusion, animal studies provide mechanistic evidence that ART can alter immune development, including thymic maturation, cytokine signaling, innate immune activity, and placental immune regulation. However, human data remain limited and should be interpreted cautiously, as many reported immune-related outcomes are rare, heterogeneous, or modest in effect size. Thus, while ART may influence immune maturation, it remains unclear whether animal mechanisms translate into clinically significant immune disease in humans. Longitudinal studies integrating immune, epigenomic, placental, and clinical endpoints are needed to define the real-world impact of ART-associated immune alterations.

#### ART and metabolic health

ART have been associated with altered metabolic health in offspring of both humans and mice, raising substantial concern regarding how these procedures influence long-term well-being [14, 83, 84, 96, 177, 178]. Metabolic outcomes are among the most intensively studied ART-associated phenotypes due to their broad consequences for systemic physiology. As noted previously, during ART the gametes and early embryos are exposed to a non-physiological environment designed to approximate, but not fully replicate, the in vivo reproductive tract. The ability of the early embryo to adapt to these artificial conditions can lead to altered developmental programming, particularly at the epigenetic level, resulting in downstream effects on fetal growth, organogenesis, and metabolic homeostasis that may not become apparent until postnatal life or adulthood.

Most mechanistic information derives from mouse studies, as ART-conceived human offspring are still relatively young and many metabolic disturbances emerge later in adulthood. In mice, multiple independent studies have shown that IVF adversely affects metabolic phenotypes, including increased body weight, elevated serum triglycerides and total cholesterol, altered HDL/LDL ratios, higher fasting glucose and insulin levels, and widespread alterations in the hepatic proteome compared to naturally conceived controls [83, 84, 179]. Notably, many of these phenotypes are sexually dimorphic: females typically exhibit more pronounced or earlier metabolic impairments, whereas males may show subtler or opposite trends.

Mouse models have also enabled detailed investigation of the mechanisms underlying these alterations. Work from Rinaudo et al has demonstrated that IVF offspring exhibit significant disruptions in hepatic and serum metabolomic profiles, with glucose metabolism emerging as one of the most affected pathways [17, 84, 96]. We have similarly shown that IVF induces elevations in serum triglycerides and cholesterol accompanied by broad changes in the hepatic proteome at 12 weeks of age, particularly in pathways related to lipid metabolism [83]. Additional studies by Zheng et al. revealed decreased enzymatic activity of glucose-metabolism pathways in ART-exposed livers, along with dysregulation of insulin signaling and glucose-homeostasis networks [180]. Other reports describe altered adipocyte size, increased hepatic lipid accumulation, impaired mitochondrial function, and reduced brown adipose thermogenic capacity in ART offspring [181, 182].

Importantly, many of these metabolic abnormalities have been directly linked to epigenetic dysregulation initiated during the pre-implantation period. IVF has been shown to alter DNA methylation at key imprinted loci such as *H19/Igf2*, *Peg3*, *Snrpn*, and *Kcnq1ot1*, genes that regulate fetal growth, trophoblast function, liver development, and metabolic homeostasis [14, 83]. ART offspring also display altered methylation of metabolic regulators, including genes involved in insulin signaling, lipid synthesis, mitochondrial biogenesis, and hepatic gluconeogenesis [83, 96]. Studies in mice indicate that these epigenetic changes persist into adulthood and correlate with the severity of metabolic phenotypes [14, 83]. Furthermore, altered histone modifications, changes in chromatin accessibility, and disrupted expression of metabolic microRNAs and tRNA-derived small RNAs have been reported in ART livers and adipose tissue, providing additional layers of epigenetic regulation influencing metabolic outcomes [183].

Human cohort studies, although more limited, also suggest that ART may influence offspring metabolism. Ceelen et al. reported elevated fasting glucose levels in pubertal IVF-conceived children, independent of subfertility, parental metabolic status, maternal age, or early-life factors such as gestational age and birthweight [184]. Similarly, Chen et al. found reduced peripheral insulin sensitivity in young adults conceived by IVF. When subjected to a high-caloric dietary challenge, these individuals displayed higher fasting glucose, impaired glucose tolerance, and insulin resistance compared to naturally conceived controls [185]. However, other studies have not detected strong associations between ART and disturbances in glucose metabolism, suggesting heterogeneity across cohorts, ART procedures, parental characteristics, and postnatal environments.

ART has also been linked to immune-related metabolic disturbances. A Swedish registry study reported a higher incidence of type 1 diabetes among offspring conceived via frozen embryo transfer (FET), an effect that may be partially explained by the higher birth weight and altered fetal-to-placental weight ratio commonly observed in FET pregnancies [186]. In addition, several studies have documented dysregulation of lipid metabolism in ART-conceived individuals. Elevated triglyceride levels, altered cholesterol profiles, and other lipid abnormalities have been observed in ART offspring and may increase the risk of later metabolic syndrome [177]. As in mice, these metabolic alterations in humans tend to emerge during late childhood or adolescence, whereas early childhood metabolic markers often remain within normal ranges [187]. Emerging reports also suggest increased systolic blood pressure, endothelial dysfunction, and altered vascular reactivity in ART-conceived children and adolescents, indicating broader cardiometabolic consequences not limited to glucose or lipid pathways.

In conclusion, mechanistic studies in mouse and other animal models provide strong evidence that ART can disrupt early developmental programming and contribute to long-term metabolic alterations. However, these findings should be interpreted cautiously in humans, where available data remain less comprehensive and many reported outcomes are rare or modest in magnitude. Thus, while accumulating clinical evidence supports potential associations between ART and offspring metabolic health, further long-term studies are needed to determine whether mechanisms identified in animal models translate into clinically significant disease in ART-conceived individuals.

#### ART and reproductive system

Among the adverse health outcomes reported in ART-conceived offspring, the effects of these technologies on their reproductive system have emerged as an area of growing interest. Gametes are highly sensitive to environmental conditions, and both oogenesis and spermatogenesis undergo extensive epigenetic remodeling that initiates during fetal development and continues postnatally. Consequently, exposure to non-physiological physical, chemical, and metabolic conditions associated with ART procedures has the potential to disrupt normal epigenetic reprogramming, with possible long-term consequences for reproductive development and function in the offspring.

Because direct assessment of reproductive tissues in humans is inherently invasive and ethically constrained, mouse models have been essential for elucidating and studying ART-induced reproductive phenotypes. In mice, Ban et al. showed that metabolic abnormalities in male offspring conceived by IVF persisted into the second generation, even in the absence of an additional metabolic stressor such as a high-fat diet [188]. Other studies have shown that ART-induced alterations in testicular development and sperm maturation impair normal sperm function, leading to adverse outcomes in the next generation. For example, recent work from our laboratory demonstrates that IVF disrupts testicular development and sperm maturation, leading to reduced sperm counts, abnormal sperm morphology, and persistent alterations in sperm DNA methylation with associated adverse metabolic and developmental outcomes in offspring sired by IVF-conceived males [81]. In parallel, Kanatsu-Shinohara et al. demonstrated in mouse ICSI models that male offspring exhibit behavioral changes and altered DNA methylation at promoter regions in testicular DNA, with evidence of transgenerational transmission of these epigenetic alterations [97]. Although direct evidence for comparable transgenerational effects in humans remains lacking, these animal studies raise important concerns regarding germline-mediated inheritance following ART.

A recent study from our laboratory demonstrated that female IVF-conceived offspring exhibit a significant reduction in ovarian reserve, accompanied by altered circulating reproductive hormone levels and widespread transcriptional changes in ovaries, oocytes, and cumulus cells [82]. These molecular and endocrine disruptions translated into reduced fecundity, as evidenced by smaller litter size when IVF-conceived females were naturally mated. To date, it remains unclear whether these female reproductive phenotypes are transmitted to subsequent generations generated by natural conception, highlighting an important gap in the field.

Human data on ART-associated reproductive outcomes remain limited, largely because much of the ART-conceived population has not yet reached puberty or advanced to an age typical of reduced reproductive success. Most available studies have focused on pubertal development, hormone levels, and early indicators of reproductive capacity. In females, Belva et al. evaluated ovarian reserves in young adult women conceived by ICSI at approximately 20 years of age and reported comparable ovarian reserve markers between ICSI- and naturally conceived individuals [189]. However, this study was limited by a relatively small cohort size, and ART protocols have evolved substantially since the conception of this population, potentially masking procedure-specific effects. Carlsen et al., using the Norwegian national ART registry, assessed the probability of achieving pregnancy among ART-conceived individuals and found a reduced likelihood of pregnancy during the follow-up period, although this was not directly linked to a clinical infertility diagnosis, they also reported largely comparable obstetric outcomes, except for a higher incidence of low Apgar scores [190]. Subsequently, Klementti et al., analyzing data from large Nordic registries across four countries, reported a higher prevalence of pubertal disorders among ART-conceived offspring [191]. While noteworthy, these findings must be interpreted cautiously, as the overall incidence of pubertal disorders has increased across populations, likely due in part to broader environmental and endocrine-disrupting exposures [191]. More recently, Feng et al., analyzing a large Chinese pediatric cohort, reported higher odds of low anti-Müllerian hormone (AMH) levels in children conceived through ART, a clinical indicator suggestive of reduced ovarian reserve later in life [192]. Finally, Catford et al. reported that males conceived via IVF or ICSI had similar sperm concentrations, total sperm counts, and total motile counts compared with naturally conceived controls, but exhibited significant differences in sperm morphology, progressive motility, gonadotropin levels, and circulating testosterone concentrations [193]. These results suggest that ART-induced disturbances in metabolic and reproductive physiology can be stably transmitted through the male germline.

In summary, mouse and other animal studies suggest that ART may have longer-term effects on reproductive function in offspring. However, human studies remain limited, and the overall impact of ART on reproductive health in ART-conceived individuals is still not well defined. Where increased risks are reported, they should be interpreted in the context of their rarity, effect size, and whether mechanisms identified in animal models translate into clinically significant reproductive disease in humans. As ART use continues to increase worldwide, long-term and multigenerational studies integrating reproductive, endocrine, and epigenomic endpoints will be essential to define potential risks and guide clinical practices that safeguard offspring reproductive health.

## Future of ART

Here we have focused on adverse health outcomes observed in ART-conceived offspring; however, most of these findings are derived from studies of traditional ART procedures. ART technologies continue to evolve rapidly, with new interventions being introduced to improve fertilization efficiency, implantation rates, and live-birth outcomes. In contrast, the identification and characterization of adverse outcomes in ART-offspring long term health with these emerging technologies have lagged. This delayed assessment limits the development of safer protocols and optimized culture conditions that could mitigate long-term risks to offspring health. Because the achievement of a live birth remains the primary benchmark of success for many clinicians and scientists, it can obscure the fact that newer ART interventions increasingly bypass natural biological barriers and coincide with critical windows of epigenetic reprogramming. Manipulations during these sensitive developmental periods have the potential to alter cellular fate decisions, organ development, and long-term physiological homeostasis, with consequences that may only manifest later in life.

One notable example is mitochondrial replacement therapy (MRT), which has been developed to prevent the transmission of pathogenic mitochondrial DNA mutations [194]. MRT has resulted in live births and can reduce the risk of severe mitochondrial disease; however, it involves extensive oocyte and embryo manipulation, and long-term metabolic, reproductive, and aging-related outcomes in MRT-conceived individuals remain largely unknown. Because human follow-up data will take decades to accumulate, well-controlled animal models are essential for evaluating long-term and multigenerational outcomes, identifying molecular and epigenetic biomarkers of risk, and distinguishing mechanistic alterations from clinically significant disease.

Similarly, emerging technologies initially developed in animal models, such as in vitro gametogenesis, involve extensive manipulation of germ cells and bypass multiple developmental checkpoints and reprogramming events that normally occur in vivo [195, 196]. Although live births have been achieved in mice, comprehensive analyses of placental development, epigenetic integrity, and long-term offspring health remain limited. Importantly, the absence of overt abnormalities at birth does not exclude subtle transcriptional, epigenetic, metabolic, or reproductive alterations that may emerge later in life.

Collectively, these observations highlight the need for longitudinal and mechanistic studies that move beyond short-term reproductive success and systematically evaluate the long-term and multigenerational consequences of established and emerging ART technologies. Well-controlled animal models will be essential for defining developmental, epigenetic, and physiological mechanisms; however, these findings should be interpreted alongside human studies that consider the rarity of some reported outcomes, the magnitude of risk, and whether experimental mechanisms translate into clinically significant disease. Such an integrated approach will be critical for guiding the responsible advancement of ART while safeguarding offspring health.

## Conclusions and clinical implications

Here, we summarize evidence suggesting that ART may contribute to adverse health outcomes in offspring, with effects originating as early as the pre-implantation stage and potentially persisting into adulthood. Although mouse models provide strong mechanistic insight, human data remain comparatively limited. Most human studies focus on relatively narrow clinical endpoints, such as circulating glucose, lipid profiles, or hormone levels, with far fewer examining tissue specific, organ level, or molecular alterations. This limited phenotyping constrains our ability to fully evaluate the long-term physiological consequences of ART and to translate mechanistic findings from animal models to humans. Expanding human studies to include longitudinal follow up, integrative multi-omic approaches, and organ specific functional assessments will be essential to define the full spectrum of ART associated outcomes and guide improvements in reproductive technologies.

At the same time, ART has enabled the birth of millions of children worldwide and remains an essential and generally safe clinical intervention, with most ART conceived individuals exhibiting normal growth and development. However, evidence from experimental models and emerging human studies suggests that embryonic manipulations during ART can influence developmental programming, including epigenetic regulation and metabolism. These observations do not undermine the clinical value of ART but highlight the importance of refining clinical practices and better understanding how specific procedures affect early development.

For clinical practice, these insights emphasize optimizing embryo culture conditions and minimizing unnecessary manipulations during pre-implantation development. Factors such as oxygen tension, culture media composition, culture duration, and embryo handling can influence embryonic metabolic and epigenetic states. Aligning in vitro conditions more closely with the physiological reproductive environment may help reduce potential long-term risks. Physicians should provide balanced counseling, reassuring patients that most ART conceived children are healthy while acknowledging that some long term molecular and physiological outcomes remain incompletely understood.

Finally, expanded longitudinal studies integrating clinical, molecular, and epigenetic analyses will be critical to determine whether subtle biological differences associated with ART translate into meaningful health outcomes across the lifespan. As ART technologies evolve, responsible innovation and rigorous evaluation will be essential to ensure that advances in reproductive medicine continue to prioritize both effectiveness and long term safety.

## Data Availability

No new data were created or analyzed in this study. Data sharing is not applicable to this article.

## Notes

**Authorship notes:** EARC is the first author.
